# Identifying classes of persons with mild intellectual disability or borderline intellectual functioning: a latent class analysis

**DOI:** 10.1186/s12888-017-1426-8

**Published:** 2017-07-17

**Authors:** Peter J. G. Nouwens, Rosanne Lucas, Nienke B. M. Smulders, Petri J. C. M. Embregts, Chijs van Nieuwenhuizen

**Affiliations:** 1 Tranzo Department, Tilburg School of Social and Behavioral Sciences, Tilburg University, Tilburg, the Netherlands; 2 Prisma Foundation, Waalwijk, the Netherlands; 3 Department of Medical and Clinical Psychology, Tilburg School of Social and Behavioral Sciences, Tilburg University, Tilburg, the Netherlands; 4 Dichterbij Innovation and Science, Gennep, the Netherlands; 5 GGzE Research Group Forensic Mental Health Care, GGzE Centre for Child & Adolescent Psychiatry and De Catamaran, Hospital for Youth Forensic Psychiatry & Orthopsychiatry, GGzE, Eindhoven, The Netherlands

**Keywords:** Mild intellectual disability, Borderline intellectual functioning, Intellectual disability, Latent class analysis, Heterogeneity, Profiles

## Abstract

**Background:**

Persons with mild intellectual disability or borderline intellectual functioning are often studied as a single group with similar characteristics. However, there are indications that differences exist within this population. Therefore, the aim of this study was to identify classes of persons with mild intellectual disability or borderline intellectual functioning and to examine whether these classes are related to individual and/or environmental characteristics.

**Methods:**

Latent class analysis was performed using file data of 250 eligible participants with a mean age of 26.1 (*SD* 13.8, range 3–70) years.

**Results:**

Five distinct classes of persons with mild intellectual disability or borderline intellectual functioning were found. These classes significantly differed in individual and environmental characteristics. For example, persons with a mild intellectual disability experienced fewer problems than those with borderline intellectual disability.

**Conclusions:**

The identification of five classes implies that a differentiated approach is required towards persons with mild intellectual disability or borderline intellectual functioning.

**Electronic supplementary material:**

The online version of this article (doi:10.1186/s12888-017-1426-8) contains supplementary material, which is available to authorized users.

## Background

Persons with a mild intellectual disability (MID; intelligence quotient (IQ) range 50–69) or borderline intellectual functioning (BIF; IQ range 70–85) are vulnerable for problems in different domains. For instance, compared to individuals with an average IQ level, persons with MID or BIF are at higher risk to develop mental health, behavioral and academic problems, and are more likely to experience socio-economic disadvantages [1, 2]. The presence of such problems implies that persons with MID or BIF are also more vulnerable for a poorer quality of life [1, 2] and even exclusion from society [3]. To avoid these risks, adequate and timely support is required that meets the support needs of these individuals [4].

Determination of the level of support needs of persons with MID or BIF is still largely based on IQ [5–7]. However, according to the American Association on Intellectual and Developmental Disabilities (AAIDD), a multidimensional perspective is needed that focuses on the additional personal and environmental characteristics which impact the support needs of persons with an intellectual disability (ID) [1, 5, 8]. Given the vulnerability of persons with MID or BIF (apart from their lower IQ), they are in particular need of such a multidimensional perspective. Indeed, according to Soenen, Van Berckelaer-Onnes and Scholte [4] persons with MID or BIF display a range of problems which cannot be identified on the basis of the IQ-criterion alone. Therefore, insight into the support needs of persons with MID or BIF is limited when the personal and environmental characteristics are not taken into account [2, 4].

In many studies persons with MID or BIF are investigated as a single group having comparable personal and environmental characteristics [5]. However, there are indications that for persons with MID or BIF differences exist in both personal and environmental characteristics. Regarding personal characteristics, persons with MID or BIF face a great diversity of comorbid psychopathologies, e.g. Autism spectrum disorders [9–12], attention deficit hyperactivity disorder [13–15], and substance use disorders [16–18]. Concerning environmental characteristics, persons with MID or BIF come from a wide range of family backgrounds [19]. For example, whereas some persons with MID or BIF come from a supportive family, others have been confronted with inconsistent parenting and even maltreatment and abuse [19, 20]. Also, besides differences in personal and environmental characteristics, there are differences in the type of professional care received by persons with MID or BIF. For example, they are not only supported within the care for people with an intellectual disability, but are also widely represented in other service systems, such as youth services, criminal justice systems, and mental health-care [21–25]. Given these differences in characteristics and professional care, it is not surprising that variability exists in the support needs within this population [4, 19].Thus, the diversity within the population of persons with MID or BIF suggests possible heterogeneity and, consequently, the need for a differentiated approach. Better insight and understanding of this heterogeneity can help in attempts to develop more individualized support programs meeting the support needs of persons with MID or BIF [19].

Until now, only a few studies have explored potential heterogeneity in persons with MID or BIF, e.g. by identifying classes [5, 22]. For example, Soenen et al. [5] identified four classes of persons with MID, based on the level of intellectual, adaptive and behavioral functioning. Furthermore, Douma, Dekker, De Ruiter, Tick, Koot and Bodfish [22] used latent class analysis and identified six unique classes of youngsters with MID or BIF with antisocial and delinquent behaviors. Until now, no studies have focused on heterogeneity in personal and environmental factors in persons with MID and with BIF, through all age groups. Therefore, the present study aims to identify unique classes of persons with MID or BIF based on personal and environmental characteristics.

## Methods

### Participants

Data were collected from 250 persons with MID (IQ range 50–69) or BIF (IQ range 70–85). Participants were individuals referred to an organization that offers long-term inpatient/outpatient care to persons with an intellectual disability, in the southern part of the Netherlands. To define the IQ scores of each participant, from their personal case file the most recent score of an IQ test, established by a certified diagnostician (e.g. a psychologist), was used. These age-related tests included, for instance, the Dutch versions of the Wechsler Preschool and Primary Scale of Intelligence, WPPSI-III-NL [26], the Wechsler Intelligence Scale for Children-Third edition, WISC-III-NL [27], and the Dutch version of the Wechsler Adult Intelligence Scale-Third edition, WAIS-III-NL [28]. Excluded from the study were persons who: (i) already received care from the care provider being investigated, (ii) were referred for short-term support (e.g. respite care or crisis care), or (iii) were referred for temporary support because they were waiting for support from another care provider.

The population of potential participants consisted of 525 persons with MID or BIF who were referred to the care provider being investigated between January 2011 and August 2012. In latent class analysis, the number of subgroups within a sample is partially dependent on sample size [29, 30]. As the number of subgroups tends to plateau with sample sizes of around 200 [30, 31], from the population of 525 persons a random sample of 250 participants was selected for the present study.

The representativeness of the sample was checked in two ways. First, the population receiving services from the care provider being investigated was compared with the population receiving services from the intellectual disability sector in the Netherlands as a whole. Using a *χ* ^*2*^ *-*test, the distribution of the various Care Intensity Packages in the clients from this care provider was compared with the distribution in the whole intellectual disability sector [32]. In the Netherlands, long-term care for persons with an intellectual disability is provided under the terms of the Exceptional Medical Expenses Act (AWBZ) and access to support requires a statement of need. Care Intensity Packages are an expression of support needs. At the time of data collection for the present study, the Care Intensity Package for an individual was determined by an independent organization (called the CIZ) on a nationwide basis, using objective criteria. No significant differences were found, indicating that the clients of the care provider being investigated were representative of the population receiving care in the intellectual disability sector in the Netherlands. Second, the representativeness of the sample of 250 respondents was examined a posteriori by comparing the gender (*χ* ^*2*^(1) = 0.32, *p* = 0.57), age (*t*(773) = 0.66, *p* = 0.51), and level of development (*χ* ^*2*^(1) = 0.01, *p* = 0.92) of participants with the initial group of eligible persons (*N* = 525). As no significant differences were found, this implies that participants in the selected sample were representative of the initial population.

The selected sample consisted of 250 persons, with significantly more males (60.8%) than females (39.2%) (*χ* ^*2*^(1) = 245.82, *p* < 0.001) and a mean age of 26.1 (*SD* 13.8; range 3–70) years. The age category with the highest frequency was 11–20 years (39.2%), followed by the age categories 21–30 (21.6%), 31–40 (13.6%), 41–50 (10.4%), 51–60 (3.6%), and 61–70 years (2.8%). A significantly higher proportion of participants had BIF (56.5%/*n* = 141) than MID (43.6%/*n* = 109) (*χ* ^*2*^(1) = 245.95, *p* < 0.001). All included persons had significant intellectual limitations and adaptive behavior problems, as based on a valid CIZ (Care Needs Assessment Center) indication. The CIZ organization uses objective criteria (on a nationwide basis) to determine whether there is a significant limitation in intellectual and adaptive functioning.

### Measures

A retrospective descriptive design was used. Data were provided by professionals from referring organizations and all the case files contained standardized information from the independent organization, the CIZ. Furthermore, most of the case files consisted of intake reports, anamnesis, support history, former support plans and evaluations, and psychological reports. These case files were analyzed using a structured case analysis system based on the Signaling List [33] and on studies by Schalock et al. [34] and Van Nieuwenhuizen et al. [35]. This resulted in a scoring list of 313 variables in the following categories: personal characteristics (subcategories: personal information, IQ level, diagnoses, level of education, support needs, anamnesis), family characteristics (subcategories: family situation and upbringing, maltreatment and abuse), contextual characteristics (subcategories: life events and treatment history). Together, the family characteristics and contextual characteristics were labeled as ‘environmental’ characteristics. The presence or absence of these characteristics was based on concrete signals or references in the case files. In addition, the person providing the information was of particular importance regarding the variables ‘problem behavior’ and ‘DSM-IV diagnosis’. For these two variables, the information had to be provided by a certified clinician (e.g. a psychiatrist or a clinical psychologist). The researchers scored the same case files separately until an inter-rater agreement of at least 80% was achieved; Cohen’s kappa was used to rate the inter-rater agreement for the dichotomous variables (*n* = 75). After the same three case files had been coded separately, the researchers did reach an inter-rater agreement of at least 80% and a Cohen’s kappa of > 0.70, indicating appropriate inter-rater agreement. After reaching a good inter-rater level, the case files analysis was performed by three researchers independently.

### Procedure

Due to the vulnerability of the persons in the study group, we deliberately selected a retrospective case analysis that did not require an active participation or action of the participants. According to the Dutch Act on Medical Treatment Agreements (article 7: 458) the following strict conditions permit a retrospective case analysis without ethical approval: (a) the study is of general interest, (b) the study cannot be conducted without the requested information, (c) the participant has not expressly objected to the provision of the data, and (d) the anonymity of the participant is guaranteed. We used a passive informed consent concerning the retrospective analyses of file data. In the present study, the noun ‘client’ refers to persons with an intellectual disability and their parents or legal guardians. Correspondingly, both for minors and for adults with an intellectual disability, clients and their parents or legal guardians were actively informed about the study by means of a tailormade brochure. The brochure also included information about the voluntariness of the study and the anonymity of the participants. Participants could object to the provision of the data. In the brochure, it was clearly stated who the client had to contact when he/she did not want to participate in this study. Three participants of the initial sample (*N* = 525) did not want to participate in the study and were therefore excluded. The names of all the participants were replaced by unique numbers to guarantee anonymity. The names of the participants and their unique numbers were registered in a separate Excel file, which was locked with a password and stored in a protected environment.

Although according to the Dutch ‘Agreement on Medical Treatment’ Act (Article 7: 458) no ethical approval was needed for the purpose of this study, several additional steps were taken regarding the ethical aspects due to the vulnerability of the participants and the level of precision that we aimed to achieve. A priori the Client Advisory Board of Prisma (i.e. the organization where the study took place) was informed. The Client Advisory Board of Prisma has a legal status on behalf of the law ‘Engagement Clients in Health Facilities’ (WMCZ in the Netherlands). The Client Advisory Board consists of parents or relatives of clients with an intellectual disability. The board is representative for the client population and focuses on the collective interests. The Client Advisory Board participated in the decision-making of this study; they confirmed the relevance of the study and gave approval for its performance. Furthermore, referring Dutch organizations for the support of disabled or chronically ill persons were informed. These independent organizations guide and support persons with intellectual disabilities and their relatives in their contacts with care providers. Professionals of these organizations were actively informed about this study and all acknowledged the importance and relevance.

### Data analysis

#### Latent class analysis

The software program Latent GOLD (version 4.5) was used to perform a latent class analysis [36]. Variables included in the latent class analysis were chosen based on the socio-ecological person-environment fit conception [6], and literature on common risk factors for persons with MID or BIF [1, 13, 20, 37–40]. As a consequence, 14 variables from the initial scoring list of 313 variables were included in the latent class analysis, divided into two higher categories: environmental variables and personal variables. The category ‘environmental variables’ consisted of two subcategories: (1) family variables and (2) contextual variables; variables were scored as either present or absent. Family variables were: divorce of parents, financial problems of parents, mental health problems of parents, harassment by primary caregiver, sexual abuse by primary caregiver, and inconsistent parenting. The contextual variables were: no informal support from friends and/or family, and difficulty with connecting to peers.

The following ‘personal variables’ were included: financial problems, daytime activity, alcohol and/or drug addiction, problem behavior, prison sentence, and the Diagnostic and Statistical Manual of Mental Disorder-IV (DSM-IV) classification [41].

Latent class analysis was used to identify classes of related persons. Briefly, latent class analysis examines the underlying structure of categorical data using probabilistic methods to assign an individual to a class, which is based on the individual’s most likely membership (see, e.g. [42]). The first step in latent class analysis is to define the number of classes that contain distinctive classes. Class membership was based on the personal variables, family variables and contextual variables described above. To determine the most appropriate latent class model, as well as the number of classes, the Akaike’s Information Criterion (AIC) with a per-parameter penalty factor of 3 (AIC3) was used. The AIC3 is a relative indicator model of fit, with lower values indicating better fit of the model to the data. The AIC3 was chosen because of the categorical variables included and due to the relatively small sample size [36, 43, 44]. The best fitting class solution was chosen based on the lowest AIC3 score, class error around 10% and bivariate residuals ≤ 4, since bivariate residuals ≥ 4 indicate a possible correlation between the variables.

#### Class membership comparison

In a second step, class membership in relation to variables other than the variables already included in the latent class analysis, was analyzed to define the different classes in more depth using SPSS 19.0, PC. Since the collected data consisted of categorical variables, a one-way analysis of variance (ANOVA) was conducted (*F*-ratio). When the overall one-way ANOVA was significant, *post-hoc* tests were performed to analyze significant differences between the different classes. The Bonferroni-correction was employed to correct for the problem of multiplicity used to indicate statistical significance.

## Results

### Latent class analysis

Regarding the latent class analysis, solutions for 1–8 classes are presented in Table 1.

**Table 1:** Fit statistics for Latent Class Analysis (*n* = 250)

Number of classes	BIC	AIC	AIC3	Class error
1	3466.31	3417.01	3431.01	0.00
2	3234.90	3132.77	3161.77	0.08
3	3245.31	3090.37	3134.37	0.11
4	3274.94	3067.17	3126.17	0.13
**5**	**3304.98**	**3044.40**	**3118.40**	**0.11**
6	3350.71	3037.30	3126.30	0.14
7	3398.35	3032.11	3136.11	0.14
8	3452.83	3033.77	3152.77	0.14

*Note.* The fit statistics suggested that the five-class solution best fitted the data

AIC3 values decreased across solutions containing 1–5 class solutions, suggesting that the five-class solution best fitted the data. The class error for the five-class solution was also lower than for the six-class solution. Furthermore, the values of the bivariate residuals were near zero and close to each other, indicating no correlation between the different variables. The outcome of these indicators indicated that a five-class solution was the best explanation for the underlying structure of the data.

### Class description

**Fig. 1:**
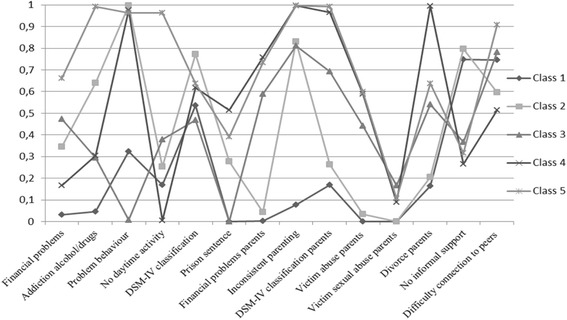
Five classes of individuals with mild intellectual disability or borderline intellectual functioning (*n* = 250) and the probability of individual, family and contextual characteristics

Figure 1 shows the probability of the presence of the personal and environmental (family and contextual) variables included in the latent class analysis for each of the five classes. Class 1 (*n* = 85) represented the largest class (34.0%); given that this was the only class characterized by more persons with MID (*n* = 55) than with BIF (*n* = 30), Class 1 was labelled as ‘Persons with mild intellectual disability’. Class 2 (*n* = 51) included 20.4% of the sample; in this class, 18 individuals had MID and 33 BIF. All persons in this class (100%) showed problem behavior, resulting in the label ‘Males with problem behavior’. Class 3 (*n* = 47) comprises 15 persons with MID and 32 with BIF; this class accounted for 18.8% of the sample and consisted of persons who had the highest score on sexual abuse by parents, and high scores on personal financial problems and financial problems of parents. As a result, Class 3 was labeled ‘Persons with material hardship and abuse by parents’. Participants in Class 4 (*n* = 37) included 14.8% of the sample and had the lowest average age; this class comprises 10 individuals with MID and 27 with BIF. Because this class was characterized by persons with high scores on problem behavior and family variables, this group was labelled ‘Male youngsters with problem behavior and family problems’. Class 5 (*n* = 30) included 12% of the sample and consisted of persons with high scores on all included variables, especially on the variable addiction to alcohol and/or drugs, resulting in the label ‘Persons with addictive problems’. This class includes 13 individuals with MID and 17 with BIF.

### Class comparison

The presence of the personal and environmental (family and contextual) variables showed a significant difference between the five classes (see Additional file 1). These class differences are described below.

Concerning the personal variables, the five classes showed a significant difference in mean age and gender distribution. More specifically, ‘Male youngsters with problem behavior and family problems’ (Class 4) were the youngest (mean age 19.1 years) and ‘Persons with material hardship and abuse by parents’ (Class 3) were the oldest (mean age 29.8 years). Regarding gender distribution, only ‘Persons with material hardship and abuse by parents’ (Class 3) consisted of more females (68%) than males (32%).

Besides differences in mean age and gender, there were also differences in the amount of financial problems and daytime activity. Although the prevalence of financial problems was highest among ‘Persons with addictive problems’ (Class 5; 67%), ‘Persons with material hardship and abuse by parents’ (Class 3) also had financial problems (51%). For daytime activity, ‘Male youngsters with problem behavior and family problems’ (Class 4) always had daytime activity (100%), whereas none of the ‘Persons with addictive problems’ (Class 5) had any daytime activity (0%).

Other significant differences were found in addiction to alcohol and/or drugs, prison sentence, and DSM-IV classifications. Addiction to alcohol and/or drugs was most prevalent in ‘Persons with addictive problems’ (Class 5; 100%) and ‘Males with problem behavior’ (Class 2; 64%). Regarding prison sentence, ‘Male youngsters with problem behavior and family problems’ (Class 4) were confronted with a prison sentence (56%) significantly more often than all other classes. Finally, the prevalence of DSM-IV classifications was highest among ‘Males with problem behavior’ (Class 2; 80%) and the lowest for ‘Persons with material hardship and abuse by parents’ (Class 3; 47%).

With regard to family variables, several significant differences were found. For instance, all parents of ‘Male youngsters with problem behavior and family problems’ (Class 4) were divorced (100%), whereas in ‘Persons with mild intellectual disability’ (Class 1) 15% of the parents were divorced. Furthermore, the prevalence of parental financial problems was highest among ‘Male youngsters with problem behavior and family problems’ (Class 4; 79%) and lowest among ‘Persons with mild intellectual disability’ (Class 1; 0%). Also, all ‘Persons with addictive problems’ (Class 5) were confronted with at least one primary caregiver classified with a DSM-IV diagnosis (100%). This was also the case for most of the ‘Male youngsters with problem behavior and family problems’ (Class 4; 96%). In contrast, of the ‘Persons with mild intellectual disability’ (Class 1) 20% had to deal with at least one primary caregiver classified with a DSM-IV diagnosis.

Differences were also found with regard to sexual abuse by primary caregiver(s). For example, 18% of the ‘Persons with material hardship and abuse by parents’ (Class 3) had been a victim of sexual abuse by their primary caregiver(s), followed by ‘Persons with addictive problems’ (Class 5; 12%). ‘Persons with mild intellectual disability’ (Class 1) and ‘Males with problem behavior’ (Class 2) had never been sexually abused by primary caregiver(s) (0%). Regarding harassment by the primary caregiver(s), the highest prevalence was found for ‘Persons with addictive problems’ (Class 5; 62%), whereas none of the ‘Persons with mild intellectual disability’ (Class 1) had been confronted with harassment by primary caregiver(s) (0%).

Regarding the contextual variables, the five classes showed a significant difference with regard to problematic connection to peers and informal support. ‘Persons with addictive problems’ (Class 5; 89%), ‘Persons with material hardship and abuse by parents’ (Class 3; 76%) and ‘Persons with mild intellectual disability’ (Class 1; 76%) had the most difficulty with connecting to peers and had no friends. Furthermore, 81% of the ‘Males with problem behavior’ (Class 2) had no informal support and for ‘Male youngsters with problem behavior and family problems’ (Class 4) this was the case for 23% of the persons.

Other differences were found related to the amount of contact with health-care providers, and the age at the moment health-care was first received. Compared with the other four classes, ‘Male youngsters with problem behavior and family problems’ (Class 4) had the most (mean 6.5 times) contact with health-care providers before referral to the care provider being investigated, and were also the youngest at the moment health-care was first received (mean age 12.5 years). Those with the least contact were ‘Persons with mild intellectual disability’ (Class 1; mean 3.7 times), and ‘Persons with material hardship and abuse by parents’ (Class 3) were the oldest at the moment health-care was received for the first time (mean age 23.9 years).

## Discussion

In many studies, persons with MID or BIF are investigated as a single group and considered to have comparable personal and environmental characteristics [5]. However, there are indications that, within these two populations, differences exist in personal and environmental characteristics. Therefore, a differentiated approach towards these individuals may be needed to acknowledge possible heterogeneity and achieve better insight into the support needs of these persons. Thus, this study investigated classes of persons with MID or BIF and identified five unique classes of persons with either MID or BIF.

In the present study, ‘Persons with mild intellectual disability’ (Class 1) had the least personal and environmental problems. Almost half of the group with MID (*n* = 53/48.6%) are part of this class, whereas this applies to a considerably smaller proportion of those with BIF (*n* = 32/22.7%). Additionally, compared to persons with MID, individuals with BIF are overrepresented in the classes with more personal and environmental problems. This is in line with Podesta and Radstaak [45], and Nouwens et al. [19] who found indications that individuals with BIF, compared to those with MID, experienced more individual and family problems. Also, whereas persons in class 1 experienced relatively few problems, many of them experienced problems with connecting to peers, resulting in the absence of friends. This might be explained by the high percentage of the DSM-IV classification of Pervasive Developmental Disorders in this class, since features of this disorder include lack of appropriate social skills, leading to problems with entering into friendships [11, 46].

In ‘Males with problem behavior’ (Class 2) and ‘Male youngsters with problem behavior and family problems’ (Class 4) personal problem behavior often co-occurred with family problems, which is in line with earlier findings [47]. In ‘Males with problem behavior’ (Class 2), the externalizing problem behavior of these persons might have placed high demands on their parents, resulting in inconsistent parenting practices [48]. In contrast, in ‘Male youngsters with problem behavior and family problems’ (Class 4) the co-occurrence of personal and family problems could be explained by a reciprocal relationship between child and family problems, with the problem behavior of the child and parental problems having a continuous and negative influence on each other, thereby reinforcing the personal and family problems [49, 50]. Although the development of child and family problems may vary between these classes, in both classes the personal and family problems need to be addressed in intervention planning.

Class 3, ‘Persons with material hardship and abuse by parents’, consisted of more women than men. The defining characteristic of this class was the high amount of sexual abuse by primary caregiver(s), corresponding with earlier findings that females (more often than males) are a victim of sexual abuse [51]. In some cases, the traumatic experience of sexual abuse by primary caregiver(s) might have resulted in a mood disorder, which was the most prevalent DSM-IV classification within this class [52].

Another finding of this study was that addiction was a major problem in ‘Persons with addictive problems’ (Class 5). This supports earlier results showing that persons with an intellectual disability are at increased risk of substance-related problems compared to persons without an intellectual disability [16]. The absence of daytime activities for all persons in Class 5 is not surprising, given that problematic substance users with MID or BIF often lack daytime activities [53, 54].

### Implications

The present results imply that a more differentiated approach is required towards persons with MID or BIF. The identification of five classes is a first step towards a better understanding of the heterogeneity within the population of persons with MID or BIF. More specifically, it provides more insight into the characteristics on different domains that together influence the support needs of these persons. The differentiation of five subgroups can be used as guidance in individual support planning. However, further ‘fine-tuning’ of the specific type of support within this ‘class’ is still required, since every individual with MID or BIF has his/her own unique characteristics.

### Strengths and limitations

There is some evidence suggesting marked similarities between people with MID and BIF [1]. In the present study, differences were found both within and between the classes for individuals with MID and BIF. Future research should investigate in more detail the differences between persons with MID and BIF. In the present study, we investigated the possibility of identifying classes within the population of persons with MID or BIF of all ages. However, some limitations of this study need to be addressed. Data were collected by examining case files. As the completeness of these files differed between the participants, some information may have been incomplete. In future research, file data could be complemented with a person’s and his/her parents’ own reporting of several life aspects, e.g. by use of a questionnaire, or conducting an interview [55].

Another limitation concerns the quality of the available file data. For example, although we used the most recent IQ data to identify persons with MID or BIF, some of the ‘most recent’ IQ data was 21 years old. Also, the IQ data originated from diverse IQ tests. Another limitation, is that due to the incompleteness of data, some evidence risk factors could not be used in the latent class analysis (e.g. education level of parents). These aspects might have influenced our results. In future research, this might be overcome by complementing file data with more recent information on the individual’s intellectual abilities, and with additional data on relevant risk factors.

A final limitation is the wide age range. Because our sample consisted of individuals aged from 3 to 70 years, the results can only offer a general understanding of the typology within this group. Future studies should focus, e.g. on specific age groups to better examine the confounding variable of the developmental period.

## Conclusions

This study is a first step towards a better understanding of the heterogeneity within the population of persons with MID or BIF. The identification of five classes of persons with MID or BIF challenges the undifferentiated approach towards these persons and seems to confirm that more differentiated individual support is needed.

## Additional file

Additional file 1: Table S2. Significant differences between the five classes of individuals with mild intellectual disability or borderline intellectual functioning (*n* = 250). (DOCX 18 kb)

## Abbreviations

AAIDD: American Association in Intellectual and Developmental Disabilities
AIC: Akaike’s information criterion
ANOVA: Analysis of variance
BIF: Borderline intellectual functioning
DSM: Diagnostic and statistical manual of mental disorders
ID: Intellectual disability
IQ: Intelligence quotient
MID: Mild intellectual disability
WAIS-III-NL: Dutch version of the Wechsler Intelligence Scale-Third edition
WISC-III-NL: Dutch version of the Wechsler Intelligence Scale for Children-Third edition
WPPSI-III-NL: Dutch version of the Wechsler Preschool and Primary Scale of Intelligence
