# Supplementary material for: Identifying classes of persons with mild intellectual disability or borderline intellectual functioning: a latent class analysis
Source: BMC Psychiatry. 2017 Jul 17;17:257. doi: 10.1186/s12888-017-1426-8 (PMC5512980; doi:10.1186/s12888-017-1426-8)
Supplement: Additional file 1: Table S2. — Significant differences between the five classes of individuals with mild intellectual disability or borderline intellectual functioning (n = 250). (DOCX 18 kb) [file 12888_2017_1426_MOESM1_ESM.docx]

**Table 2** Significant differences between the five classes of individuals with mild intellectual disability or borderline intellectual functioning (*n*=250)

|  | Class 1 (*n*=85)  ‘Persons with mild intellectual disability’ | Class 2 (*n*=51)  ‘Males with problem behavior’ | Class 3 (*n*=47)  ‘Persons with material hardship and abuse by parents’ | Class 4 (*n*=37)  ‘Male youngsters with problem behavior and family problems’ | Class 5 (*n*=30)  ‘Persons with addictive problems’ | *F* | Significant differences |
| --- | --- | --- | --- | --- | --- | --- | --- |
|  | % | % | % | % | % |  |  |
| *Individual variables* |  |  |  |  |  |  |  |
|  |  |  |  |  |  |  |  |
| Average age | *M* = 27.1 | *M* = 24.7 | *M* = 29.8 | *M* = 19.1 | *M* = 28.0 | 3.75^*^ | 1, 3 > 4 |
| Gender |  |  |  |  |  | 7.42^**^ | 1, 2, 4 > 3 |
| Male | 64.0 | 80.0 | 32.0 | 70.0 | 53.0 |  |  |
| Female | 36.0 | 20.0 | 68.0 | 30.0 | 47.0 |  |  |
| Intellectual Disability |  |  |  |  |  | 5.42^**^ | 2, 3, 4 > 1 |
| Mild intellectual disability | 62.0 | 35.0 | 32.0 | 27.0 | 43.0 |  |  |
| Borderline | 38.0 | 65.0 | 68.0 | 73.0 | 57.0 |  |  |
| Financial problems | 2.0 | 34.0 | 51.0 | 19.0 | 67.0 | 20.39^**^ | 2, 3, 5 > 1  5 > 2  3, 5 > 4 |
| No daytime activity | 19.0 | 22.0 | 37.0 | 0.0 | 100.0 | 34.12^**^ | 5 > 1, 2, 3, 4  3 > 4 |
| Alcohol/drugs addiction | 3.0 | 64.0 | 31.0 | 31.0 | 100.0 | 30.48^**^ | 2, 3, 4, 5 > 1  2 > 3, 4  5 > 2, 3, 4 |
| Problem behavior | 31.0 | 100.0 | 0.0 | 100.0 | 100.0 | 136.58^**^ | 1, 2, 4, 5 > 3  2, 4, 5 > 1 |
| DSM-IV classification**^a^** | 53.0 | 80.0 | 47.0 | 59.0 | 63.0 | 3.66^*^ | 2 > 1, 3 |
| Most present DSM-IV classification | Pervasive Developmental Disorder (31.3) | Pervasive Developmental Disorder (19.2) | Mood Disorder  (11.8) | ADHD (18.9) | Substance-related disorder  (20.0) | - | - |
| Prison sentence | 0.0 | 26.0 | 0.0 | 56.0 | 38.0 | 26.38^**^ | 2, 4, 5 > 1, 3  4 > 2 |
| Outplacement | 28.0 | 38.0 | 11.0 | 52.0 | 59.0 | 4.64^*^ | 4, 5 > 1 |
|  |  |  |  |  |  |  |  |
| *Family variables* |  |  |  |  |  |  |  |
|  |  |  |  |  |  |  |  |
| Divorced parents | 15.0 | 18.0 | 60.0 | 100.0 | 64.0 | 36.07^**^ | 3, 4, 5 > 1, 2  4 > 3, 5 |
| Financial problems parents | 0.0 | 3.0 | 63.0 | 79.0 | 77.0 | 57.69^**^ | 3, 4, 5 > 1, 2 |
| DSM-IV diagnosis primary caregiver(s) | 20.0 | 26.0 | 68.0 | 96.0 | 100.0 | 27.98^**^ | 3, 5, 5 > 1, 2 |
| Victim harassment primary caregiver(s) | 0.0 | 3.0 | 48.0 | 59.0 | 62.0 | 31.50^**^ | 3, 4, 5 > 1, 2 |
| Victim sexual abuse primary caregiver(s) | 0.0 | 0.0 | 18.0 | 9.0 | 12.0 | 4.89^*^ | 3 > 1, 2 |
|  |  |  |  |  |  |  |  |
| *Contextual variables* |  |  |  |  |  |  |  |
|  |  |  |  |  |  |  |  |
| No informal support | 77.0 | 81.0 | 35.0 | 23.0 | 29.0 | 11.79^**^ | 1, 2 > 3, 4, 5 |
| Difficulty with connection to peers | 76.0 | 58.0 | 77.0 | 53.0 | 89.0 | 4.08^*^ | 5 > 2, 4 |
| Average number of health care providers | *M* = 3.7 | *M* = 5.1 | *M* = 4.2 | *M* = 6.5 | *M* = 6.0 | 12.82^**^ | 2, 4, 5 > 1  4, 5 > 3 |
| Average age at first contact with health care provider | *M* = 18.7 | *M* = 14.3 | *M* = 23.9 | *M* = 12.5 | *M* = 18.9 | 4.07^*^ | 3 > 2, 4 |

^a^ According to APA [40].

^*^*P*<0.01, ^**^*P*<0.001.

.
